# Supplementary material for: Resolving the full spectrum of human genome variation using Linked-Reads
Source: Genome Res. 2019 Apr;29(4):635–45. doi: 10.1101/gr.234443.118 (PMC6442396; doi:10.1101/gr.234443.118)
Supplement: Supplemental Material [file supp_gr.234443.118_Supplemental_longranger-2.2.zip › longranger-2.2.tar.bz2!/lr2.2-public/lib/stan/cmdstan/stan/doc/api/html/ddv__vari_8hpp_source.html]

Stan: src/stan/agrad/rev/internal/ddv\_vari.hpp Source File


|  |  |
| --- | --- |
|  | Stan  2.5.0  probability, sampling & optimization |


- Main Page
- Related Pages
- Namespaces
- Classes
- Files

- File List
- File Members

All Classes Namespaces Files Functions Variables Typedefs Enumerations Enumerator Friends Macros Pages

- src
- stan
- agrad
- rev
- internal

ddv\_vari.hpp

Go to the documentation of this file.

1 #ifndef STAN\_\_AGRAD\_\_REV\_\_INTERNAL\_\_DDV\_VARI\_HPP

2 #define STAN\_\_AGRAD\_\_REV\_\_INTERNAL\_\_DDV\_VARI\_HPP

3

4 #include <stan/agrad/rev/vari.hpp>

5

6 namespace stan {

7  namespace agrad {

8

9  class op\_ddv\_vari : public vari {

10  protected:

11  double ad\_;

12  double bd\_;

13  vari\* cvi\_;

14  public:

15  op\_ddv\_vari(double f, double a, double b, vari\* cvi) :

16  vari(f),

17  ad\_(a),

18  bd\_(b),

19  cvi\_(cvi) {

20  }

21  };

22

23  }

24 }

25 #endif

stan::agrad::op\_ddv\_vari

**Definition:** ddv\_vari.hpp:9

stan::agrad::op\_ddv\_vari::cvi\_

vari \* cvi\_

**Definition:** ddv\_vari.hpp:13

vari.hpp

stan::agrad::vari

The variable implementation base class.

**Definition:** vari.hpp:28

stan::agrad::op\_ddv\_vari::bd\_

double bd\_

**Definition:** ddv\_vari.hpp:12

stan::agrad::op\_ddv\_vari::op\_ddv\_vari

op\_ddv\_vari(double f, double a, double b, vari \*cvi)

**Definition:** ddv\_vari.hpp:15

stan::agrad::op\_ddv\_vari::ad\_

double ad\_

**Definition:** ddv\_vari.hpp:11


---

[ Stan Home Page ]

*© 2011–2014,
Stan Development Team.*
